# Wide use of broad-spectrum antibiotics in very low birth weight infants with spontaneous focal intestinal perforation—is it really justified?

**DOI:** 10.1007/s15010-024-02257-2

**Published:** 2024-04-18

**Authors:** Sarina K. Butzer, Kirstin Faust, André Oberthuer, Charlotte Kleindiek, Benjamin Kuehne, Christoph Haertel, Katrin Mehler

**Affiliations:** 1 Division of Pediatric Infectious Diseases, Department of Pediatrics, Faculty of Medicine, University of Cologne, University Hospital Cologne, Kerpener Straße 62, 50937 Cologne, Germany; 2 Department of Pediatrics, University of Lübeck, Lübeck, Germany; 3 Department of Pediatrics, University of Würzburg, Würzburg, Germany; 4 German Neonatal Network (GNN), Lübeck, Germany; 5 Division of Neonatology, Department of Pediatrics, Faculty of Medicine, University of Cologne, University Hospital Cologne, Cologne, Germany

**Keywords:** Very low birthweight infants, Focal intestinal perforation, Antimicrobial stewardship, Carbapenem, Vancomycin

## Abstract

**Purpose:**

Very low birth weight (VLBW) infants are at a risk of spontaneous focal intestinal perforation (FIP). Treatment includes supportive care, antibiotics, and drainage with/without surgery. Broad-spectrum antibiotic agents like carbapenems are applied frequently, although their use is not well-supported by the limited evidence of causal pathogens. We hypothesize that the use of carbapenems may not be necessary in VLBW infants with FIP. Our primary objective was to evaluate the antimicrobial use in VLBW infants with FIP in a cohort of the German Neonatal Network (GNN). The secondary objective was to characterize a subset in detail as a benchmark for future targets of stewardship.

**Methods:**

Data on VLBW infants with FIP was collected prospectively within the GNN, a collaboration of 68 neonatal intensive care units (NICU). With regards to the primary objective, patient characteristics and antimicrobial treatment were extracted from the predefined GNN database. To address our secondary objective, an additional on-site assessment of laboratory and microbiological culture results were performed.

**Results:**

In the GNN cohort, 613/21,646 enrolled infants (2.8%) developed FIP requiring surgery. They were frequently treated with carbapenems (500/613 (81.6%)) and vancomycin (497/613 (81.1%)). In a subset of 124 VLBW infants, 77 (72.6%) had proof of gram-positive bacteria in the abdominal cavity, coagulase-negative staphylococci (CoNS) predominantly. Despite the low prevalence of gram-negative bacteria (*n* = 6 (4.8%)), the combination of meropenem and vancomycin was prescribed most frequently (*n* = 96 (78.0%)).

**Conclusion:**

The use of carbapenems as broad-spectrum antimicrobials agents might not be justified in most VLBW infants with FIP. Knowledge on the development of the neonatal gut microbiota, local resistance patterns and individual microbiological findings should be taken into consideration when implementing antimicrobial stewardship programs (ASPs).

## Purpose

Very low birth weight (VLBW) infants are at increased risk of spontaneous focal intestinal perforation (FIP). FIP is characterized by a localized intestinal perforation with no or minimal tissue inflammation. Unlike necrotizing enterocolitis (NEC), which typically occurs later in VLWB infants [1–3], FIP is commonly diagnosed within the first 10 days of life. Another differentiating aspect is the characteristic excessive inflammation occurring in infants with NEC [3, 4]. Both, FIP and NEC are presumed to have multifactorial causes [5–8].

Along with extreme prematurity, the use of non-steroidal anti-inflammatory drugs and steroids are key factors for FIP pathophysiology [9, 10]. Standard of care treatment includes cessation of enteral food or medication, supportive care, intravenous antibiotics, peritoneal drainage and surgery [11]. Clinical practice guidelines published by the Infectious Disease Society of America suggest that broad-spectrum antibiotics may be useful in patients with FIP [12]. However, the evidence on FIP-causative pathogens and targeted antibiotic treatment strategies in VLBW infants with FIP is scarce. In Germany, two guidelines address antimicrobial therapy in neonates [13, 14] but clear recommendations for VLBW infants with FIP are not given.

In this study we aimed to evaluate the antimicrobial use in VLBW infants with FIP in multiple tertiary care neonatal intensive care units (NICU) participating in the German Neonatal Network (GNN). The primary objective of this study was to evaluate empiric antibiotic therapy in VLBW infants diagnosed with FIP. The secondary objective was to characterize microbiological findings in a detailed subset analysis of patients treated at three GNN centers. We hypothesized that, based on clinical findings, laboratory results and pathogenic spectra the use of carbapenems may not be necessary in VLBW infants with FIP. This provides an opportunity to improve treatment concepts within the context of antimicrobial stewardship.

## Methods

### Study design and patients

Data on VLBW infants with FIP were prospectively collected within the GNN, a collaboration of 68 tertiary care NICUs in Germany. The GNN is an observational data collection initiative focusing on long-term development of VLBW infants. This collaboration started patient recruitment on January 1, 2009, and has been ongoing continuously. Patient characteristics and postnatal treatment was recorded on data sheets at the participating centers by study nurses or supervising physicians. Subsequently, data sheets were sent to the study center post-discharge. The GNN study was approved by the institutional review board at the University of Lübeck and at all participating study sites. Written informed consent was obtained by the infants’ guardians.

This study employed a retrospective design. Due to data retrieval from a predesigned database, available information was predetermined. With regards to our primary objective, data extraction from the GNN database was performed by applying the following inclusion criteria: (a) infants with birth weight less than 1500 g and (b) documented FIP requiring surgery. The last patient was included on December 31, 2021. Clinical features that were drawn from the database included birthday, gestational age, birth weight, sex, multiple births, antenatal steroids, 5-min Apgar score, inotrope support within 24 h of life, GNN site, occurrence of clinical sepsis or culture-proven sepsis, FIP and antibiotic prescriptions. Standard protocols dictate blood culture collection prior to any antibiotic exposure. However, a review of actual practice has not been conducted.

To investigate our secondary objective, we conducted additional on-site audits in a subset of VLBW infants with FIP from three participating GNN sites (Cologne, Lübeck, Würzburg). For this purpose, patient records were reviewed retrospectively from March 2016 until March 2023. Included were (a) infants with a birth weight < 1500 g and (b) documented FIP irrespective of whether they underwent surgical intervention. Excluded were infants with either only gastric or rectal perforation, malrotation or ileal atresia. Depending in center-specific policies, electronic and/or paper-based information was used in each patient. Various parameters were collected, including birthday, gestational age, birth weight, sex, laboratory results, histopathology, intraabdominal cultural results, data of multiplex-bacterial and -fungal PCR and antimicrobial treatment in timely association with FIP. Intraabdominal cultural collection comprised cultures obtained during intraabdominal catheter placement and during surgery. Standard protocols specify microbiological culture collection prior to antimicrobial treatment, if feasible. Microbiological data on subsequent surgeries were not included into the secondary objective analysis.

### Definitions

Sepsis was defined according to the criteria of the national infection surveillance system (NEO-KISS) as either culture-negative clinical sepsis (clinical and laboratory signs of sepsis and clinician’s decision to treat with antibiotics for at least 5 days) or culture-confirmed sepsis [15]. Furthermore, we differentiated inflammatory from non-inflammatory FIP. Inflammatory FIP was recorded as soon as one of the following criteria were met (1) greyish/mottled skin appearance and capillary refill time > 2 s, (2) painful palpation of the abdomen and (3) CRP ≥ 10 mg/L and/or IL6 ≥ 40 ng/L. FIP without inflammation was documented when the infant presented with (1) normal skin appearance and regular capillary refill time plus (2) soft abdomen after drainage plus (2) CRP < 10 mg/L and/or IL6 < 40 ng/L. This classification was not predefined and is not yet validated within any other cohort. We established this differentiation in anticipation of future antimicrobial stewardship efforts, with the aim of potentially identifying risk groups and implementing risk-adapted treatment.

### Statistical analysis

Statistical analysis was performed with IBM® SPSS Statistics (version 28). For the primary and secondary objectives, normal distribution was determined by the Kolmogorov–Smirnov-test. Furthermore, proportions of nominal variables were compared using Pearson’s χ^2^ test and Fisher’s exact test for both objectives. Median values were compared non-parametrically by two-sided Mann–Whitney *U*-tests. Investigating the primary objective, multivariate analyses were performed by applying logistic regression models including known confounders which might have an impact on the clinical decision to treat with a carbapenem or vancomycin, i.e. gestational age, small for gestational age, sex, multiple births, antenatal steroids, 5-min Apgar score, inotrope support within 24 h of life, GNN site, year of birth, clinical sepsis or culture-proven sepsis and FIP [16]. *p*-Values < 0.05 were considered significant.

## Results

### Patient characteristics and antimicrobial treatment

Of 21,646 enrolled VLBW infants from 68 German NICUs, 613 presented with FIP requiring surgery (2.8%) (Table 1). Median gestational age was 24 5/7 weeks, median weight at birth was 665 g. With regards to our primary objective, infants with FIP were also diagnosed with clinical sepsis (*n* = 379/613 (61.8%)) or any culture-confirmed sepsis (*n* = 192/613 (31.3%)), including 150/613 (24.5%) infants with gram-positive sepsis, 49/613 (8.0%) infants with gram-negative sepsis and 17 (2.8%) infants with *Candida* spp. sepsis. Table 2a describes the exposure rate with different anti-infective agents stratified according to diagnosis of culture-positive and -negative sepsis, respectively.

**Table 1:** Patient characteristics of 613 VLBW infants with focal intestinal perforation and detailed information on a subset of 124 VLBW infants with or without surgery

	Total cohort *n* = 613	Subset *n* = 124
Median gestational age in weeks (IQR)	24 5/7 (23 4/7–25 6/7)	24 1/7 (23 1/7–25 3/7)
Median weight at birth in grams (IQR)	665 (525–805)	622 (490–739)
Sex		
Male (%)	375 (61.2)	67 (54.0)
Female (%)	238 (38.8)	57 (46.0)
Median days to pneumoperitoneum (IQR)		5 (2–8)
Median days to surgery (IQR)		6 (5–10)
Conservative approach (%)		19 (15.3)
Median days from pneumoperitoneum to surgery		1 (0–3)
Clinical symptoms (*n* = 113)		
Distended but soft abdomen (%)		72 (63.7)
Abdominal pain with peritonism (%)		41 (36.3)
Drainage (*n* = 92)		
Clear, amber-colored		35 (38.0)
Bilious		16 (17.4)
Cloudy		6 (6.5)
Stool		25 (27.0)
No secretion		10 (10.9)
Classification (*n* = 105)		
Inflammatory FIP		48 (45.7)
Non-inflammatory FIP		46 (43.8)
Not to classify		7 (6.7)
Others^a^		4 (3.8)
Laboratory results		
Median IL6 [ng/L] at the time of pneumoperitoneum (IQR) (*n* = 36)		68 (15.3–835.8)
Median CRP [mg/L] at the time of pneumoperitoneum (IQR) (*n* = 87)		2 (0.6–5.5)
Median IL6 [ng/L] at the time of surgery (IQR) (*n* = 29)		208 (69–3157)
Median CRP [mg/L] at the time of surgery (IQR) (*n* = 97)		7 (2–32.8)
Macroscopic findings (*n* = 93)		
Intestinal perforation only		71 (76.3)
Inflammatory bowl		15 (16.1)
Necrotizing bowl		7 (7.5)
Microscopic findings (*n* = 78)		
Inflammation		48 (61.5)
Hemorrhage		16 (20.5)
Ectatic lymphatic/blood vessels		10 (12.8)
Necrosis		16 (20.5)
Status until first discharge (*n* = 124)		
Alive (%)		97 (78.2)
Died (%)		17 (13.7)
Died within 14 days after pneumoperitoneum (%)		6 (4.8)
Lost to follow-up (%)		10 (8.1)

^a^Not to distinguish from NEC

**Table 2:** (a) Administered antibiotic therapy in the total cohort of VLBWI with FIP, (b) administered antibiotic therapy in a subset of 124 VLBWI with FIP

(a)			
	Culture-negative sepsis *n* = 421 (%)	Culture-positive sepsis *n* = 192 (%)	Total *n* = 613 (%)
Aminoglycosides	346 (82.2)	168 (87.5)	514 (83.8)
Gentamicin	219 (52.0)	113 (58.9)	332 (54.2)
Tobramycin	139 (33.0)	59 (30.7)	198 (32.3)
Carbapenems*	329 (78.1)	171 (89.1)	500 (81.6)
Meropenem*	284 (67.5)	153 (79.7)	437 (71.3)
Imipenem	60 (14.3)	29 (15.1)	89 (14.5)
Vancomycin*	326 (77.4)	171 (89.1)	497 (81.1)
Cefotaxim	236 (56.1)	103 (53.6)	339 (55.3)
Cefuroxim	60 (14.4)	25 (13.2)	85 (13.9)
Ceftazidim	74 (17.6)	36 (18.8)	110 (17.9)
Piperacillin/tazobactam*	89 (21.1)	63 (32.8)	152 (24.8)
Ampicillin/sulbactam	59 (14.0)	34 (17.7)	93 (15.2)
Metronidazol	121 (28.7)	52 (27.1)	173 (28.2)
Teicoplanin	57 (13.5)	27 (14.1)	84 (13.7)
Linezolid*	20 (4.8)	21 (10.9)	41 (6.7)
Fosfomycin	13 (3.1)	9 (4.7)	22 (3.6)
Fluconazol	109 (25.9)	66 (34.4)	175 (28.5)
Liposomal amphotericin B*	44 (10.5)	33 (17.2)	77 (12.6)

(b)	
	Subset *n* = 124
Missing data	*n* = 1
Median days of antibiotic treatment (IQR)	11 (2–72)
Antibiotic monotherapy first-line (%)	15 (12.2)
Ampicillin/sulbactam	2
Piperacillin/tazobactam	6
Vancomycin	6
Meropenem	1
Antibiotic combination therapy first-line (%)	108 (87.8)
Ceftazidim + vancomycin	3
Piperacillin/tazobactam + vancomycin	6
Meropenem + vancomycin	96
Piperacillin/tazobactam + meropenem	2
Ampicillin + meropenem	1
Antibiotic combination therapy at any time point (%)	117 (95.1)
Switch of antibiotic substances during therapy (%)	20 (16.3)

*Significant differences between non-sepsis vs. sepsis are indicated: Carbapenems *p* = 0.001, Meropenem *p* = 0.002, Vancomycin *p* < 0.001, Piperacillin/tazobactam *p* = 0.003, Linezolid *p* = 0.006, Amphothericin B *p* = 0.03

**Fig. 1:**
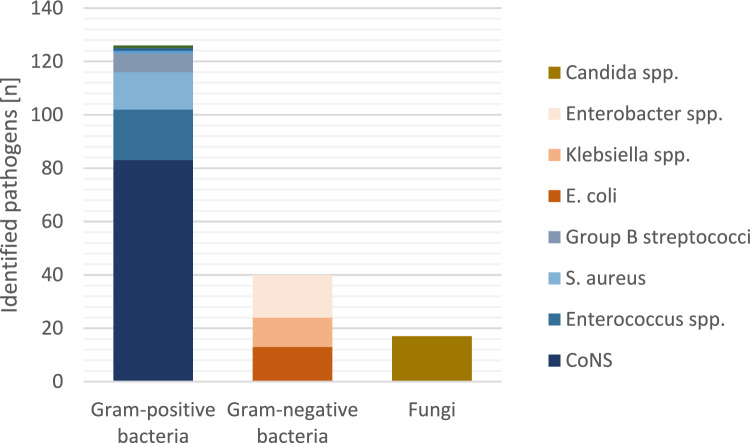
Blood-culture isolated pathogens in 613 VLWB infants with FIP

As illustrated in Fig. 1, CoNS were the most common isolated pathogens within blood-cultures (*n* = 83/613 (13.5%)), followed by *Enterococcus* spp. (*n* = 19/613 (3.1%)), *Enterobacter* spp. (*n* = 16/613 (2.6%)), *Staphylococcus aureus* (*n* = 14/613 (2.3%)), *E. coli* (*n* = 13/613 (2.1%)), *Klebsiella* spp. (*n* = 11/613 (1.8%)). Less common were Group B streptococci (*n* = 7/613 (1.1%)), *Serratia* spp. (*n* = 3/613 (0.5%)) and *Pseudomonas* spp. (*n* = 1/613 (0.2%)). However, independent of concomitant sepsis diagnosis, VLBW infants with FIP were frequently exposed to meropenem/imipenem (culture-negative sepsis: *n* = 329/421 (78.1%); culture-positive sepsis: *n* = 171/192 (89.1%)) and vancomycin (culture-negative sepsis: *n* = 326/421 (77.4%); culture-positive sepsis: *n* = 171/192 (89.1%; Table 2a). In line with this, the diagnosis of clinical sepsis did not remarkably change prescription patterns of carbapenems or vancomycin, specifically meropenem/imipenem (no clinical sepsis: *n* = 171/234 (73.1%); clinical sepsis: *n* = 329/379 (86.8%)) and vancomycin (no clinical sepsis: *n* = 167/234 (71.4%); clinical sepsis: *n* = 330/379 (87.1%)). For the whole GNN cohort, the multivariate logistic regression analysis with “meropenem or imipenem use” as dependent variable revealed an association between the diagnosis of FIP and the use of meropenem or imipenem (OR 6.58, 95% CI: 5.23–8.27, *p* < 0.001). Confounding variables were gestational age, small for gestational age, sex, multiple birth, antenatal steroids, 5-min Apgar score, inotrope support within 24 h of life, GNN site, year of birth, culture-confirmed sepsis and FIP. In a similar model using clinical sepsis as confounder, FIP was also associated with meropenem or imipenem use (OR 6.49, 95% CI: 5.14–8.19, *p* < 0.001). Likewise, the use of vancomycin was associated with FIP diagnosis adjusted for culture-confirmed sepsis (OR 4.03, 95% CI: 3.21–5.06, *p* < 0.001) or clinical sepsis (OR = 3.89, 95% CI: 3.08–4.91, *p* < 0.001), respectively.

### Low prevalence of gram-negative bacteria in abdominal cavities of VLBW infants with FIP

With regard to out secondary objective, a detailed audit of clinical and laboratory data was performed in 124 VLBW infants with FIP in three tertiary care GNN sites (Table 1). Pneumoperitoneum occurred at a median interval of 5 days, with surgery being performed up to 3 days thereafter. Notably, 21 (19.9%) were outborn and transferred for surgery. An intraperitoneal drainage was established in 92 infants, 19 (15.3%) were managed without surgery. Most infants presented with abdominal distension, the number of infants with non-inflammatory vs. inflammatory FIP were similar. Evaluated inflammatory markers comprised Interleukin-6 (IL6) and C-reactive protein (CRP). Median IL6 increased from 68 to 208 ng/L from time of pneumoperitoneum to time of surgery, which was not significant (*p* = 0.55), while CRP increased significantly from 2 to 7 mg/L (*p* = 0.008). Median time from birth to recognition of FIP was significantly longer in infants with signs of inflammation (7 vs. 4 days, *p* < 0.05). The smaller subset of patients with macroscopic necrotic tissue was diagnosed at a median age of 3 days. Histopathological results were available in 78 cases, inflammation was the most frequent finding (*n* = 48 (61.5%)). Notably, necrotic tissue was identified in 16 patients (20.5%), with only two being older than 14 days and only one exhibiting the aforementioned macroscopic impression of necrosis. A second surgery was done in 31/105 infants (29.5%), with a large proportion of infants presenting with a second FIP at a different localization (*n* = 15/105 (14.3%)), while 12/105 patients (11.4%) had suture dehisces after the first surgery. Surgeons diagnosed NEC in three cases of second-look surgery, while one infant had a resection of necrotic tissue.

As outlined in Table 2b, most infants received an antibiotic combination therapy (*n* = 117/123 (95.1%)). A combination therapy was commenced in 108/123 (87.8%) infants, most frequently with meropenem and vancomycin (*n* = 96/123, 78.0%). Monotherapy was started in 15 infants, most frequently with beta-lactam/beta-lactamase-inhibitors (*n* = 8/123 (6.5%)). Median duration of antimicrobial treatment was 11 days (IQR 2–72). Infants with inflammatory FIP were treated longer than infants with non-inflammatory FIP [16 days (IQR 10–23 days) vs 9 days (IQR 7–14 days); *p* < 0.001].

**Fig. 2:**
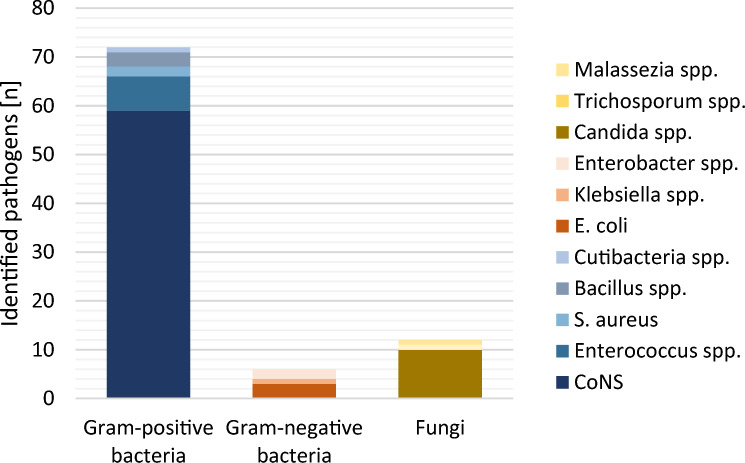
Proportion of pathogens identified within the abdominal cavity of a subset of 124 VLBW infants with FIP

Abdominal cultures were collected in 106 patients either during abdominal catheter placement and/or during surgery and revealed 96 positive results. As shown in Table 3 and Fig. 2, CoNS were most frequently found in 59/96 (55.7%) patient cultures, while growth of gram-negative bacteria (*n* = 6/96 (5.7%)) and fungi (*n* = 13/96 (12.3%)) was less common. When multiple organisms were identified (*n* = 15/96 (14.2%)), CoNS were evident in all cultures. Another common combination included fungi (*n* = 8/15 (53.3%)), *Candida* spp. in particular (*n* = 7/15 (46.7%)). In corresponding blood cultures, two infants were found to have evidence of bacteria. One exhibited KNS (while having *Enterococcus* spp. and *Candida* spp. in the abdominal cultures), while another patient showed the presence of *Bifidobacteria* spp., which were not found in abdominal cultures. Among all identified intraabdominal gram-negative organisms, one was not susceptible to empirical antibiotic therapy. In this case, the patient received Vancomycin only, gram-negative organisms were covered additionally after receiving intraabdominal proof of *E. coli*. Exclusive carbapenem sensitivity was evident in three infants with proof of *Enterobacteriaceae*.

**Table 3:** Identified pathogens within the abdominal cavity

	Identified by drainage	Identified by drainage and surgery	Subset *n* = 124 (%)
Microbial analysis not done			18
No pathogen identified	25		38
Identified species			96
Gram-positive bacteria	34	12	77 (72.6)
CoNS	27	11	59 (55.7)
*Enterococcus* spp.	4	1	7 (6.6)
*Staphyloccocus aureus*	1		2 (1.9)
*Bacillus* spp.	2		3 (2.8)
*Cutibacterium* spp.			1 (0.9)
Gram-negative bacteria			6 (5.7)
*Escherichia coli*	1	1	3 (2.8)
*Klebsiella* spp.			1 (0.9)
*Enterobacter* spp.			2 (1.9)
Fungi			13 (12.3)
*Candida* spp.	1	1	10 (9.4)
*Aspergillus* spp.			1 (.9)
*Trichosporum* spp.			1 (0.9)
*Malessezia* spp.			1 (0.9)
Polymicrobial pathogen identification			
2 pathogens			15 (14.2)
≥ 3 pathogens			3 (2.8)

### Outcome

Of 124 patients, 107 survived until discharge or transferal to another hospital. None of the deaths were attributed to FIP, which was assessedby the attending physicians. When comparing antimicrobial regimens with regard to use of meropenem, mortality rate did not differ (deaths in meropenem-treated patients 13/97 (13.4%) vs. deaths in non-meropenem-treated patients 4/27 (14.8%); *p* = 0.64).

## Discussion

Carbapenems, vancomycin and aminoglycosides are the most common used antibiotic agents for VLBW infants with FIP in German NICUs, regardless of concomitant diagnosis of culture-confirmed or clinical sepsis. Microbiological findings of the audited subset of 124 VLBW infants with FIP showed a predominance of CoNS within the abdominal cavity, only a minor proportion presented with gram-negative bacteria. Among the identified Enterobacteriaceae, only three were susceptible to carbapenems exclusively. Consequently, broad-spectrum antibiotics such as carbapenems may not be justified for these patients.

FIP is a serious complication in VLBW infants with varying incidences depending on the population studied. Our cohort presented a rate of 2.8%, comparable to other previously published VLBW infants groups [17]. The onset of FIP in our subset of 124 infants was at 5 days of life (median) with abdominal distension as the key clinical hallmark present in all cases. As discrimination between FIP and NEC may not always be easy [1–3], a proportion of infants within our cohort presented with macroscopic and/or microscopic signs of inflammation or necrotizing tissue. Infants with macroscopic signs of inflammation were diagnosed later than infants without inflammation. Yet, necrotizing tissue was found very early in a smaller subset of patients. Furthermore, histopathological signs of inflammation were seen in 44 VLBW infants. In four cases, clear discrimination between NEC and FIP was not possible, as only one was diagnosed later than 14 days of life, while necrotic tissue was described histopathologically in three cases. With regards to the primary and secondary objective of our study, knowledge on the developing gut microbiota is crucial for future targeted treatment approaches of infants with FIP. The neonatal gut microbiota is highly dynamic and evolves rapidly within the first few weeks of life [18, 19]. While *Staphylococcus* spp. and *Streptococcus* spp. dominate within the first week of life, the abundance of *Enterococci* spp., gram-negative bacteria, and strict anaerobes like *Bifidobacterium* spp. increases afterwards [20–24]. Presence of fungi is also known [8, 22, 25]. Since there is a variability in the occurrence of FIP, the timing of sample collection varied in our cohort. Still, 75% of all intraabdominal cultures were gathered within the first week of life. Literature on microbiological findings within the group of VLBW infants and FIP is scarce. A study from 2020 analyzed the incidence of bacteremia/fungemia in infants with FIP or NEC but found only 3/32 positive cases, i.e. two with CoNS, one with *E. coli* [26]. Abdominal culture results were not analyzed. The decreased abundances of Firmicutes and Bacteroidetes and the increased relative abundances of Gamma-Proteobacteria appear to be consistent with NEC-associated dysbiosis, while no dysbiotic feature has been linked to FIP so far [4, 21, 27]. Our abdominal culture-based microbiological results obtained either from abdominal drainage catheter placement or during surgery mainly revealed gram-positive bacteria, especially CoNS. A minority presented with gram-negative organisms. Exclusive carbapenem sensitivity was evident in three infants with intraabdominal proof of *Enterobacteriaceae*. Notably, the proportion of identifiable fungal species within the abdominal cavity is higher than what is typically found in blood cultures, likely due to the site of culture collection.

Evidence of intraabdominal pathogens alone do not provide conclusive evidence of invasive infection, however, they might help guiding antimicrobial treatment.

Antimicrobials are frequently prescribed in VLBW infants due to the difficulty of distinguishing infection from non-infectious conditions as symptoms in neonates are not specific [28]. Furthermore, a significant number of infants in our cohort received broad-spectrum antibiotics independent of sepsis diagnosis. This special group of patients is highly vulnerable and at increased risk for severe infections, resulting in significant concerns regarding the potential severe outcomes if left undertreated. As a result, broad-spectrum agents are often preferred in VLBW infants with possible bacterial infection. ASPs aim to optimize the appropriate and judicious use of antimicrobial agents. The importance of ASPs in the NICU becomes apparent considering previously published data, showing not only gut microbiota disruption but also increased mortality and morbidities following non-rational use of antimicrobials [29–35]. Feasibility and safety of such programs in the NICU were shown before [36, 37]. In Germany, pediatric infectious diseases specialization is still evolving, and corresponding expertise is not comprehensively available nationwide. Consequently, ASP initiatives are currently implemented in only a limited number of NICUs. Our data emphasize the urgent need for expanding expertise in this area.

While empiric antibiotic treatment for early-onset sepsis typically involves a beta-lactam agent combined with an aminoglycoside, the management of late-onset sepsis varies from center to center in Germany. Common antibiotic regimens include a combination of a glycopeptide with an aminoglycoside, or a third-generation cephalosporin with a glycopeptide, or a beta-lactam beta-lactamase inhibitor. Treatment decisions also depend on the focus of infection. Moreover, in Germany, weekly screenings of VLBW infants are conducted to assess colonization status, which may influence the choice of empiric treatment [38]. Literature on the use of antimicrobial substances within the setting of FIP is extremely limited [39, 40], underlining the need for more studies to allow for improved future antibiotic treatment concepts. More data is available on infants with NEC. Still, a recently published review concludes, current evidence does not sufficiently favor any specific antibiotic regimen for the treatment of NEC [39, 41, 42]. In our total cohort of 613 VLWB infants as well as in the smaller subset of 124 infants with FIP, antimicrobial therapy with meropenem and vancomycin were the most common prescribed agents. Considering the aforementioned microbiological findings within our cohort and the conclusions drawn from knowledge of the development of the neonatal gut microbiota, we suggest that broad-spectrum antimicrobials like carbapenems are not indicated in the majority of these patients. Instead, piperacillin/tazobactam might be a suitable option. Interpretation of the increased cultural identification of CoNS in our cohort remains challenging though. While sample contamination frequently leads to false-positive identification of CoNS, conversely, they form a significant part of the developing gut microbiota in VLBW infants at that age, potentially elevating the risk of infection and indeed being relevant in this group of patients.

Limitations of this analysis comprises the trial design, data collection within the prospective GNN trial allows associations only. Furthermore, documented antimicrobial treatment includes a wide period of time from birth until discharge. We lack reliable data on the prescribed antimicrobial therapy specifically related to FIP for the total cohort. Another point of discussion is the differentiation between NEC and FIP. Achieving a definitive differentiation between NEC and FIP in a clinical setting is not entirely feasible. Inflammatory FIP and NEC are particularly hard to discriminate, also the here applied definitions inflammatory vs. non-inflammatory FIP lack established standards. However, for our assessment, the timing of suspected infection appears to be more relevant.

## Conclusion

Empirical therapy for VLBW infants with FIP should target typical pathogenic bacteria found in the neonatal intestinal flora. Given the potential hazards linked to broad-spectrum carbapenems, medical professionals should thoroughly assess the necessity of these substances. In contrast, treatment with vancomycin might be justified in a substantial number of these infants. ASPs are urgently needed to facilitate a more rational utilization of antibiotics without causing harm.

## Data Availability

Data not publicly available.

## Abbreviations

GNN: German Neonatal Network
VLBW: Very low birth weight
NICU: Neonatal intensive care units
FIP: Focal intestinal perforation
CoNS: Coagulase-negative staphylococci
ASP: Antimicrobial stewardship program
